# Angiotensin AT_2_ Receptor Stimulation Alleviates Collagen-Induced Arthritis by Upregulation of Regulatory T Cell Numbers

**DOI:** 10.3389/fimmu.2022.921488

**Published:** 2022-07-08

**Authors:** Bettina Sehnert, Veronica Valero-Esquitino, Georg Schett, Thomas Unger, Ulrike Muscha Steckelings, Reinhard Edmund Voll

**Affiliations:** ^1^ Department of Rheumatology and Clinical Immunology, Medical Center – University of Freiburg, Faculty of Medicine, University of Freiburg, Freiburg, Germany; ^2^ Center for Cardiovascular Research, Charité-Medical Faculty Berlin, Berlin, Germany; ^3^ Department of Internal Medicine 3 - Rheumatology and Immunology, Friedrich-Alexander-University Erlangen-Nürnberg (FAU) and Universitätsklinikum Erlangen, Erlangen, Germany; ^4^ Deutsches Zentrum Immuntherapie, Friedrich-Alexander-University Erlangen-Nürnberg (FAU) and Universitätsklinikum Erlangen, Erlangen, Germany; ^5^ Cardiovascular Research Institute Maastricht (CARIM), Maastricht University, Maastricht, Netherlands; ^6^ Institute of Molecular Medicine (IMM) – Department of Cardiovascular & Renal Research, University of Southern Denmark, Odense, Denmark; ^7^ Center for Chronic Immunodeficiency (CCI) Freiburg, Medical Center – University of Freiburg, Faculty of Medicine, University of Freiburg, Freiburg, Germany

**Keywords:** collagen-induced arthritis (CIA), renin–angiotensin system, angiotensin AT2 receptor agonist, cytokines, regulatory T cells

## Abstract

The angiotensin AT_2_ receptor (AT_2_R) is a main receptor of the protective arm of the renin-angiotensin system and exerts for instance anti-inflammatory effects. The impact of AT_2_R stimulation on autoimmune diseases such as rheumatoid arthritis (RA) is not yet known. We investigated the therapeutic potential of AT_2_R-stimulation with the selective non-peptide AT_2_R agonist Compound 21 (C21) in collagen-induced arthritis (CIA), an animal model for inflammatory arthritis. Arthritis was induced by immunization of DBA/1J mice with collagen type II (CII). Prophylactic and therapeutic C21 treatment alleviates arthritis severity and incidence in CIA. Joint histology revealed significantly less infiltrates of IL-1 beta and IL-17A expressing cells and a well-preserved articular cartilage in C21- treated mice. In CIA, the number of CD4^+^CD25^+^FoxP3^+^ regulatory T (Treg) cells significantly increased upon C21 treatment compared to vehicle. T cell differentiation experiments demonstrated increased expression of FoxP3 mRNA, whereas IL-17A, STAT3 and IFN-gamma mRNA expression were reduced upon C21 treatment. In accordance with the mRNA data, C21 upregulated the percentage of CD4^+^FoxP3^+^ cells in Treg polarizing cultures compared to medium-treated controls, whereas the percentage of CD4^+^IL-17A^+^ and CD4^+^IFN-gamma^+^ T cells was suppressed. To conclude, C21 exerts beneficial effects on T cell-mediated experimental arthritis. We found that C21-induced AT_2_R-stimulation promotes the expansion of CD4^+^ regulatory T cells and suppresses IL-17A production. Thus, AT_2_R-stimulation may represent an attractive treatment strategy for arthritis.

## Introduction

Rheumatoid arthritis (RA) is an inflammatory autoimmune disease of the joints (1). Driving forces in the pathogenesis of RA and experimental arthritides are cytokines such as tumor necrosis factor alpha (TNF-alpha), interleukin-1 alpha/beta (IL-1 alpha/beta), interleukin-6 (IL-6) and interleukin-17 (IL-17) (2). Moreover, T helper cell subsets that were investigated in RA patients and experimental models either exacerbate or inhibit the inflammatory process. While T helper 17 (Th17) cells promote autoimmunity and inflammation, regulatory T cells (Tregs) attenuate autoimmune diseases and maintain immune homeostasis (3–5).

The Th17-derived pro-inflammatory cytokine IL-17A is detectable in the synovial fluid and synovium of RA patients (6, 7). Experiments in collagen-induced arthritis (CIA) showed that overexpression of IL-17 in the knee joints of collagen type II (CII)-immunized mice promoted arthritis and aggravated joint destruction, whereas in IL17-deficient mice arthritis was markedly attenuated (8, 9). In models of arthritis, a shift towards Treg cells alleviated inflammatory responses. The adoptive transfer of CD4^+^CD25^+^ cells significantly reduced arthritis symptoms in CII-immunized mice (10), while depletion of CD4^+^CD25^+^ T regulatory cells aggravated the clinical symptoms in murine CIA (11).

Immunotherapeutics for targeting IL-17A have been developed for the treatment of RA and psoriatic arthritis (PsA). Clinical phase II studies showed promising results in both RA and PsA (12), however, phase III clinical trials were only successful in PsA (13). Enhancing Treg numbers by low-dose IL-2 might also represent a powerful intervention in RA therapy (14). Despite great advances in RA treatment, the development of new anti-rheumatic agents is necessary in order to offer treatment options to those patients that either inadequately respond to currently available treatments or suffer from adverse drug effects (15, 16).

Activation of the renin–angiotensin-system is mainly known for hypertension, sodium and water retention, oxidative damage, inflammation and fibrosis (17). It is acknowledged that these classical features are mediated through angiotensin AT_1_ receptor (AT_1_R) signaling (18). Moreover, several groups described a relationship between autoimmune diseases and AT_1_R actions (19–21). In contrast, stimulation of the angiotensin AT_2_ receptor (AT_2_R) leads to tissue protection and confers inhibition of inflammation, fibrosis, and apoptosis (22–24). AT_2_R stimulation inhibits key elements of inflammation in different cell types and conditions. For instance, inhibitory effects on the activity of cyclooxygenase-2 (COX-2), NF-kappaB and the tyrosine and serine phosphorylation of signal transducers and activators of transcription (STATs) have been described (25–28). The synthesis of the orally active, specific and selective, first non-peptide small molecule AT_2_R agonist, Compound 21 (C21), brought a new momentum to AT_2_R research, because it not only significantly facilitated experimental *in vivo* studies on AT_2_R actions, but it also made the AT_2_R a drug target of potential clinical interest (29). C21 is currently in Phase II/III clinical development for COVID-19 and idiopathic pulmonary fibrosis (https://www.vicorepharma.com).

The present study aimed to examine the therapeutic potential of AT_2_R stimulation by C21 in CIA. C21 treatment alleviates clinical and histological features of CIA. We suggest that increased numbers of regulatory T cells measured in the spleens of CIA mice contribute to these beneficial effects. This concept is supported by our *in vitro* data demonstrating a shift towards Treg differentiation by C21.

## Material and Methods

### Reagents

Mouse splenocytes and naïve T helper cells were cultured in RPMI 1640 (Life Technologies GmbH, Darmstadt, Germany), 2 mM glutamine, 10% heat-inactivated fetal bovine serum (Life Technologies GmbH, Darmstadt, Germany), 100 IU/mL penicillin plus 100 μg/mL streptomycin and 50 µM beta-mercaptoethanol.

The following recombinant cytokines and antibodies were used: mIL-2 and mTGF-beta (R&D, Wiesbaden, Germany), mIL-6 and mIL-12 (Biozol, Eching, Germany), mIL-23 (ebiosciences, Frankfurt, Germany); anti-CD3 antibody (clone 145-2C11) and anti-CD28 antibody (clone 37.51) (BD, Heidelberg, Germany), anti-IL-4 antibody and anti-mouse IFN-gamma antibody (Biozol, Eching, Germany),

Compound 21 (C21) was kindly provided by Vicore Pharma (Gothenburg, Sweden).

### Mice

Male DBA/1J and female C57BL/6J mice were purchased from Janvier Laboratories, Le Genest-Saint-Isle, France. Mice were maintained under conventional housing with 5 mice per cage. The mice were maintained under controlled 12 h light/12 h dark cycles. Blood collection and mouse handling were reduced to a minimum to avoid stress. The animal studies were approved by the local governmental commission for animal protection of Freiburg (AZ G14/099).

### C21 Treatment in CIA

Seven-to 9-week-old DBA/1J mice were intradermally immunized once at the base of the tail with bovine type II collagen (bCII) (Chondrex, MD, Biosciences) emulsified in complete Freund’s adjuvant (CFA) (DIFCO, Detroit, MI, USA) as previously described (30). Mice were randomly divided in three groups: Group 1 received PBS (vehicle), group 2 received C21 at a dose of 0.1 mg/kg bodyweight in PBS and group 3 was treated systemically with etanercept (ETN) at a dose of 2 mg/kg bodyweight, 3 times per week. Mice received daily intraperitoneal (i.p.) injections of vehicle or C21. We used two protocols to examine the efficacy of C21 in CIA: (1) prophylactic treatment started simultaneously with CII immunization; PBS, n=14; C21, n=13. (2) early therapeutic treatment started at day 20 after CII immunization; PBS, n= 45; C21, n=42; ETN, n=20. Mice were regularly inspected for signs of swollen paws and scored from two independent investigators in a blinded fashion. The arthritis score was graded on a scale of 0–4 for each paw (31). Each limb was graded, scores summed, yielding in a maximum score of 16 per mouse. The clinical arthritis score for each group was expressed as mean ± SEM of scoring points per group. The area under the curve (AUC) of the arthritis score was calculated from day 18 to day 47 of CIA. The cumulative incidences were calculated for mice that had a mean arthritis score ≥1.

### Joint Histology

Hind paws were examined for infiltration of inflammatory cells and cartilage damage. In brief, mice were euthanized by CO_2_, hind paws were dissected, fixed in 4% paraformaldehyde (PFA) for 24 hours and decalcified in 10% EDTA, 100 mM Tris pH 7.5 for 2 weeks at room temperature under constant shaking (32). Paraffin embedded hind limb joints were stained with hematoxylin (Sigma, Taufkirchen, Germany), TRAP (Sigma), or toluidine blue (Sigma). The 4 µm sections were assessed by applying scores for inflammation (grades 0-3), cartilage damage (grades 0-3), and bone destruction (grades 0-3) by two independent investigators in a blinded fashion (32). Photographs were taken with an ApoTome microscope Zeiss (Zeiss, Oberkochen, Germany), original magnification 5x, using the Zeiss software Zen 2012.

### Immunohistochemical Stainings

Immunohistochemical (IHC) staining for IL-17A (# 91649, Abcam, Cambridge, UK) and Il-1 beta (Abcam, Cambridge, UK) (# 9722, Abcam, Cambridge, UK) were performed using the peroxidase-based EnVision+ System-HRP (DAB) Kit (Kit K4010, Dako, Hamburg, Germany). Joint sections were deparaffinized, rehydrated and subsequently an antigen retrieval was performed according to the manufacturers’ instructions. Endogenous peroxidases were blocked with 3% H_2_O_2_ in 60% methanol. Non-specific binding of the antibodies was reduced by blocking with 3% BSA in PBS/0.5% Tween20. Primary antibodies (5 µg/ml diluted in blocking buffer) or rabbit polyclonal IgG for isotype control (# 27472, Abcam, Cambridge, UK) were applied. The anti-rabbit peroxidase labeled polymer was subjected to cover specimen and incubated for 30 min at RT. Development was performed with 3,3′-diaminobenzidine 4-HCl (DAB) for 7 min. Sections were counterstained with Meyer’s Hematoxylin. At the end, the specimens were mounted using an aqueous-based mounting medium and a cover slip. Photographs were taken with an ApoTome microscope Zeiss (Zeiss, Oberkochen, Germany) using the Zeiss software Zen 2012. The histological sections were assessed by semiquantitative analysis. 15 randomly chosen sections per group from three independent CIA experiments were used and a minimum of 3 fields per section were analyzed. The area of DAB positive cells (IL-1 beta^+^ and IL-17A^+^ cells) was obtained by importing into ImageJ (v.1.47) for image analysis (33). By setting the threshold to appropriate values, DAB positive cells were displayed. The values were expressed as percentage DAB-positive areas (IL-1 beta^+^ and IL-17A^+^ cells) (highlighted area/non-highlighted area x 100).

### Blood Collection

The blood of CIA mice was collected in serum vacutrainer tubes (BD, Heidelberg, Germany), centrifuged for 10 min at 13.000 × g and stored at -20°C.

### Anti-CII IgG ELISA

Serum levels of total anti-CII IgG antibodies, anti-CII IgG2a and anti-CII IgG2b subclasses were measured by ELISA. Microtitre plates were coated with 10 μg/ml native bCII in PBS, blocked with 2% bovine serum albumin (BSA)/PBS and then incubated with mouse sera in 1% BSA/PBS. Bound IgG was detected by incubation with horseradish-conjugated goat anti-mouse IgG, IgG2a and IgG2b, respectively (Biozol, Eching, Germany). For color development ABTS substrate was added, and the optical density (OD) was read at 405 nm (ELISA Reader Infinite F50; Tecan, Crailsheim, Germany).

### Matrix Metalloproteinase-3 (MMP-3) ELISA

The Quantikine Mouse Total MMP3 Immunoassay (R&D, Wiesbaden, Germany) was used to detect MMP3 in sera of CIA mice. The assay was performed according to the manufacturer’s instructions and the ODs were determined using a microplate reader (ELISA reader F50; Tecan, Crailsheim, Germany) at 450 nm. Data of the standard were fit to a four parameter logistic (4-PL) curve. The calibration curve was used to calculate the concentrations of the CIA serum samples in ng/ml.

### Monocyte-Chemoattractant Protein 1 (MCP-1) ELISA

The MCP-1 mouse uncoated ELISA kit (Thermo Fisher scientific, Darmstadt, Germany) was used to detect MCP-1 in sera of CIA mice. The assay was performed according to the manufacturer’s instructions and the ODs were determined using a microplate reader (ELISA reader F50; Tecan, Crailsheim, Germany) at 450 nm. Data of the standard were fit to a four parameter logistic (4-PL) curve. The calibration curve was used to calculate the concentrations of the CIA serum samples in ng/ml.

### Transmigration Assay

*In vitro* transmigration assay trough cytokine-activated mesenteric lymph node endothelial cells (*mlEND* cells) (34) was performed as previously described (31). *mlENDs* were treated with *mlEND* culture medium (control group) or with C21 at a dose of 1 µM. This dose of C21 was used in previous *in vitro* experiments and elicited anti-inflammatory effects (26, 35, 36). Neutrophils were isolated as described in (37). Migrated neutrophils were counted in a hematocytometer (38).

### *In Vitro* Differentiation of Naïve T Cells

Naïve T cells from C57BL/6J mice were enriched by negative selection using the mouse CD4^+^CD25^-^CD62L^+^T cells Isolation Kit II (Miltenyi, Biotec, Bergisch Gladbach, Germany). The purified CD4^+^CD25^-^CD62L^+^ T cells were sedimented and resuspended to 1x10^6^ cells/ml in culture medium. 100 µl of the cell suspension was seeded in each well of a 96-well flat-bottom plate. The plate was pre-coated overnight with an anti-CD3 antibody (5 µg/ml, 100 µl/well in bicarbonate buffer pH 9.6). In the presence of respective cytokines and antibodies, naïve T cells were differentiated into Th1, Th17 or Treg subpopulations. The final volume per well was 200 µl. For Th1 differentiation: soluble anti-CD28 antibody (2.5 µg/ml), mIL-2 (20 ng/ml), mIL-12 (20 ng/ml), anti-IL-4 antibody (5 µg/ml); for Th17 differentiation: soluble anti-CD28 antibody (2.5 µg/ml), mTGF-beta 1 ng/ml), mIL-6 (20 ng/ml), mIL-23 (50 ng/ml), anti-IL-4 antibody (5 µg/ml); anti-IFN gamma antibody (5 µg/ml); for Treg differentiation: soluble anti-CD28 antibody (2.5 µg/ml), mTGF-beta (1 ng/ml), mIL-2 (20 ng/ml), anti-IL-4 antibody (5 µg/ml); anti-IFN-gamma antibody (5 µg/ml). The cells were incubated for further 5 days at 37°C and 5% CO2.

Cells were daily treated with C21 at a final concentration of 1 µM or vehicle (medium). Each individual Th differentiation experiment was set-up as technical quadruplicates from n=3 (RNA) and n=4 (intracellular stainings) C57BL/6J mice (biological replicates). T cell differentiation was analyzed by flow cytometry or gene expression analysis.

### Intracellular FACS Staining

Splenocytes were isolated from CIA mice and total number of live splenic cells was counted using an electronic cell counter (Countess, Invitrogen, Germany). CD4, CD8 and CD25 surface molecules were stained with FITC-conjugated anti-mouse CD4 (Biolegend #100509), PeCy5-conjugated anti-mouse CD8 (Biolegend # 100709) and PE-conjugated anti-mouse CD25 (Biolegend # 101903) antibodies for 15 minutes at 4°C in the dark. Intracellular staining with anti-FoxP3-APC was performed according to the manufacturer’s instructions (anti-FoxP3 antibodies human and mouse, # 130-093-013, Miltenyi, Biotec, Bergisch Gladbach, Germany). Cells were measured on a FACSCalibur flow cytometer (BD Biosciences) and data were analyzed using KALUZA software (BeckmanCoulter, Krefeld, Germany). List mode data files of 200,000 gated viable lymphocytes were collected for each sample. Lymphocytes were gated based on SSC/FSC characteristics. FoxP3^+^ cells were gated on CD4^+^CD8^-^CD25^+^ events and the frequency of CD4^+^CD25^+^FoxP^+^ cells in respect to total living splenocytes was used to calculate the absolute numbers of CD4^+^CD25^+^FoxP^+^ cells (absolute cell number = frequency of total (%) of CD4^+^CD25^+^FoxP^+^ cells multiplied by the number of total splenocytes divided by 100). Due to the relatively high variation of absolute cell number in between experiments, the summarized data from all experiments were expressed as relative changes (in %) compared to the PBS group of the respective experiment, which was regarded as 100%.

For intracellular staining of CD4^+^FoxP3^+,^CD4^+^IFN-gamma^+^ and CD4^+^IL-17A^+^ of *in vitro* differentiated naïve CD4^+^ T cells, cells were fixed and permeabilized using Fix/Perm solutions (BD, Heidelberg, Germany) according to the manufacturer’s instructions. After permeabilization, cells were incubated with anti-mouse CD4-PerCP-Cy5.5 (eBiosience #42-0042-80), anti-mouse IL-17A-PE (Biolegend #506903), FITC-conjugated anti-mouse IFN-gamma antibody (Biolegend # 505805) and AF647-conjugated anti-mouse FoxP3 (BD, Heidelberg, Germany #560402). Labeled cells were washed twice with FACS buffer (PBS/2% FCS) and then analyzed using a Coulter Gallios flow cytometer (BeckmanCoulter, Krefeld, Germany). List mode data files were collected and results expressed as percentages of the gated populations.

### RNA Extraction and Real-Time Quantitative PCR

Five days after *in vitro* polarization of T helper cells, total RNA was purified using the RNeasy Mini Kit (Qiagen, Hilden, Germany). 150 ng total RNA was reversely transcribed using the SuperScript^®^ VILO™ cDNA Synthesis Kit according to the manufacturers’ instructions (Life technologies, Frankfurt, Germany) in a total volume of 20 µl. 1 µl of cDNA was used in a 20-μl reaction volume containing 10 μl Sybr Select (Life technologies, Frankfurt, Germany) with 192 nM of each primer (mFoxP3 forward GGCCCTTCTCCAGGACAGA, reverse GCT GAT CAT GGC TGG GTT GT; mIL-17 forward CCT CAA AGC TCA GCG TGT CC, reverse GAG CTC ACT TTT GCG CCA AG;, mIFN-gamma forward AAC GCT ACA CAC TGC ATC T, reverse GAG CTC ATT GAA TGC TTG G; mHPRT forward GTT AAG CAG TAC AGC CCC AAA, reverse AGG GCA TAT CCA ACA AAC TT). HPRT was chosen as a reference gene transcript. The experimental data (CT values) were generated and analyzed by an Applied Biosystems real-time PCR instrument StepOne Plus. Relative changes in gene expression were analyzed by the [2^(-ΔΔCT)]. CT values were normalized using HPRT and the PBS group was used as calibrator. Data were presented as x-fold change to PBS group. PCRs were set-up in triplicates.

### Statistical Analysis

Statistical analyses were performed using the GraphPad Prism 9 software. All data were expressed as mean ± SEM. In the case of normal distribution, unpaired Student’s t-test (two groups) and one-way ANOVA (three groups) followed by *post-hoc* analysis were used. Not normally distributed populations were analyzed by Mann–Whitney U test. Repeated measurements were analyzed with two-way ANOVA (three groups) followed by *post-hoc* analysis. The Kaplan-Meier-Estimator was used to show differences in incidences of experimental arthritis performing log rank (Mantel-Cox). Statistical significance was defined as p ≤ 0.05 and rating of statistical significance was defined as * = P ≤ 0.05; ** = P ≤ 0.01; *** = P ≤ 0.005; **** = P ≤ 0.0001.

## Results

### Prophylactic and Early Therapeutic Use of C21 Attenuates CIA

**Figure 1:**
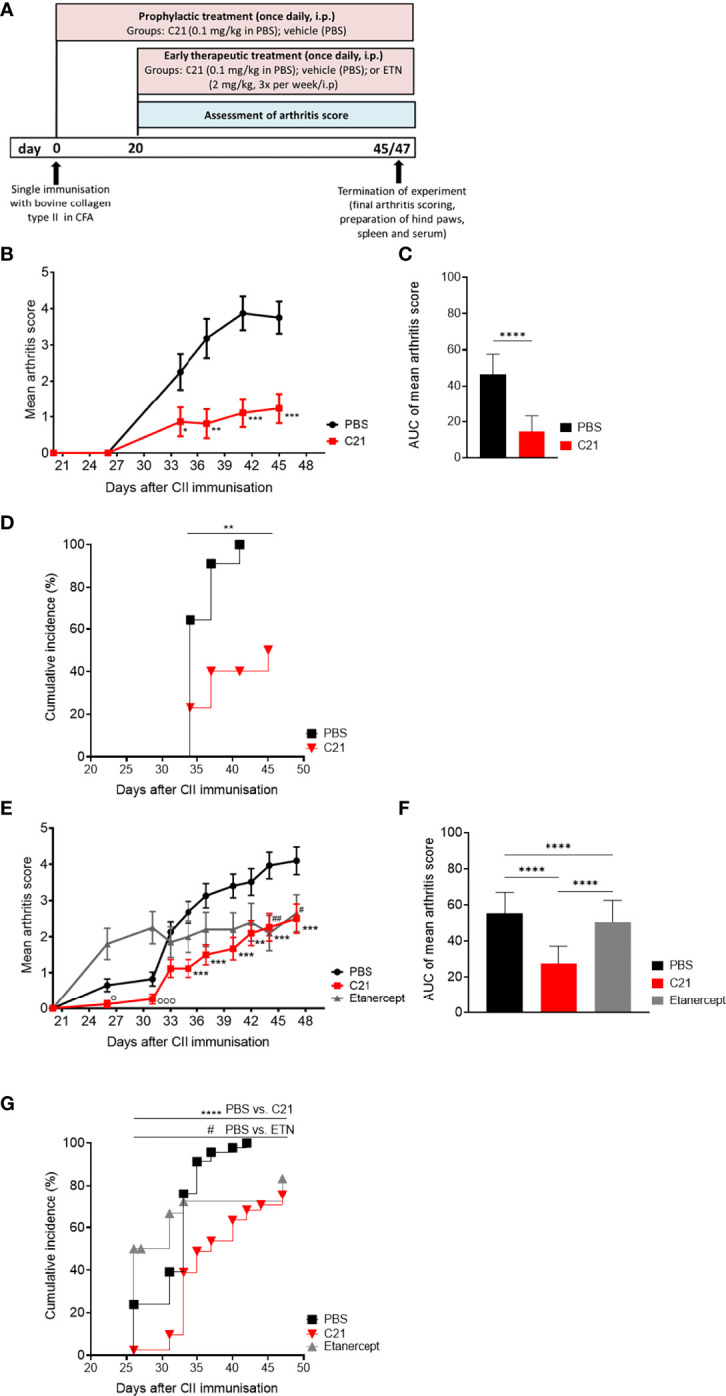
C21-induced AT2-receptor (AT_2_R) stimulation attenuates clinical manifestations and incidence of CIA by prophylactic and early therapeutic treatment. **(A)** C21 treatment protocols in collagen-induced arthritis (CIA): joint inflammation was induced by a single intradermal immunization with bovine collagen type II (CII) in complete Freund’s adjuvants (CFA) at day 0. Prophylactic treatment started at day 0 and continued until day 45 with intraperitoneal (i.p.) injections of C21 once daily at a dose of 0.1 mg/kg bodyweight. The control group received PBS (vehicle). Early-therapeutic treatment started at day 20 and continued until day 47. A third group was included receiving the soluble TNF-receptor-Ig fusion protein etanercept (ETN; 2 mg/kg; 3 times a week). Arthritis score was assessed by visual inspection of paw swelling from day 20 until the end. Hind paws, spleens and sera were taken for further analyses. **(B–D)** Data of prophylactic treatment. PBS (n=14), C21 (n=13): **(B)** Mean arthritis score. P*<0.05, P**<0.01, P***<0.001. **(C)** Area under curve analysis of mean arthritis score. P****<0.0001. **(D)** Cumulative incidence of CIA. P**<0.01. **(E–G)** Data of therapeutic treatment. PBS (n= 45), C21 (n=42), ETN (n=20). **(E)** Mean arthritis score. P**<0.01 C21 *vs*. PBS, P***<0.001 C21 *vs*. PBS, P^#^<0.05 ETN *vs*. PBS, P^##^<0.01 ETN *vs*. PBS, P°<0.05 C21 *vs*. ETN, P^ooo^<0.001 C21 *vs*. ETN. **(F)** Area under curve analysis of mean arthritis score. P****<0.0001. **(G)** Cumulative incidence of CIA mice. P****<0.0001 C21 *vs*. PBS, P^#^<0.05 C21 *vs*. ETN. Values shown are the mean ± SEM. P values were calculated by two-way ANOVA with Bonferroni’s correction **(B, E)**, unpaired Student’s t-Test **(C)** or by one-way ANOVA with Bonferroni’s correction **(F)**. Kaplan-Meier-method was used to calculate differences in incidences of experimental arthritis performing log rank (Mantel-Cox) test **(D, G)**.

The immunization of susceptible mouse strains with heterologous collagen type II (CII) emulsified in Freund’s adjuvant provokes an autoimmune response that attacks the joints (39). We investigated the clinical impact of selective AT_2_R stimulation with the non-peptide AT_2_R-agonist C21 on CIA. The C21 treatment schedule is illustrated in **Figure 1A**. First, the effect of C21 on CIA was investigated in a prophylactic treatment setting. For this purpose, mice were treated once daily with an intraperitoneal injection of C21 at a dose of 0.1 mg/kg bodyweight. The first dose was applied simultaneously with CII immunization **(** **Figure 1A**, prophylactic treatment). Severity of arthritis was assessed by scoring the joint swelling of hind and front paws and expressed as mean arthritis score. First signs of paw swelling developed around day 27. Prophylactic C21 treatment significantly reduced severity of arthritis from day 34 to day 45 compared to vehicle (PBS) **(** **Figure 1B** **)**. The area-under-curve analysis of the mean arthritis score is presented in **Figure 1C** and summarizes the strong anti-arthritic effect of C21 compared to PBS. The cumulative incidence of arthritis was estimated by a log rank (Mantel-Cox) and revealed that C21-treated CIA mice developed arthritis with a lower incidence rate compared to vehicle-treated mice **(** **Figure 1D** **)**.

Next, we examined whether AT_2_R stimulation could be beneficial when C21 is administered once arthritis has already developed. Therefore, C21 treatment started at day 20 after CII immunization and continued for additional 27 days (**Figure 1A**, early therapeutic treatment). At this stage pathogenic anti-CII autoantibodies are already present and contribute to CIA pathology (40). As treatment control, an additional group of mice was included receiving the soluble TNF-receptor-Ig fusion protein etanercept (ETN; 2mg/kg, 3 times/week, i.p.), a TNF-alpha antagonist approved for the treatment of RA and known to potently decrease incidence and severity of CIA (41, 42).

In **Figure 1E**, the arthritis scores of the early therapeutic treatment are presented. CII-immunization resulted in a significant progression of arthritis in all three groups from day 20 (score 0) to day 47 (scores: PBS: 4.098 ± 0.382, **** = P ≤ 0.0001; ETN: 2.650 ± 0.509, **** = P ≤ 0.0001; C21: 2.5 ± 0.4003, **** = P ≤ 0.0001). Between day 35 and day 47, C21 administration significantly reduced the severity of arthritis in comparison to vehicle-treated mice **(** **Figures 1E, F** **)**. The inhibition of arthritis by C21 was strongest in the early phase from day 20 to day 37, whereas TNF neutralization did not reduce arthritis score in the early phase of CIA and reached a significantly lower score than the PBS group only at day 44. The slope of increase in the arthritis score was similar between C21- and PBS-treated groups at the later phase of arthritis between day 40 and 47, however with still significantly lower arthritis scores in the C21-treated mice **(** **Figures 1E, F** **)**. At the end of the experiment, therapeutic efficacy of C21 was comparable to that of ETN. During the first 10 days of treatment, C21 was superior to ETN whereby arthritis severity was significantly reduced on day 26 and day 31 compared to ETN-treated mice. The area-under-curve analysis of the mean arthritis scores supports the strong anti-arthritic effect of C21 compared to PBS and ETN **(** **Figure 1F** **)**. Furthermore, the occurrence of symptoms was delayed **(** **Figure 1G** **)** and the incidence rate of arthritis was significantly lower in C21-treated mice compared to the vehicle- and ETN-treated groups **(** **Figure 1G** **)**. Hence, C21 appears to exert its therapeutic effects mainly in the early phase of CIA, whereas TNF antagonists exert their effects in the later phase of CIA.

Altogether, C21 improved clinical manifestations and incidence of CIA. C21 did not only exert protective effects in a prophylactic intervention trial suggesting an inhibition of the production of pathogenic autoantibodies against CII, but it was also effective as an early stage treatment of established CIA.

### C21 Reduces Inflammation and Prevents Cartilage Loss in CIA

**Figure 2:**
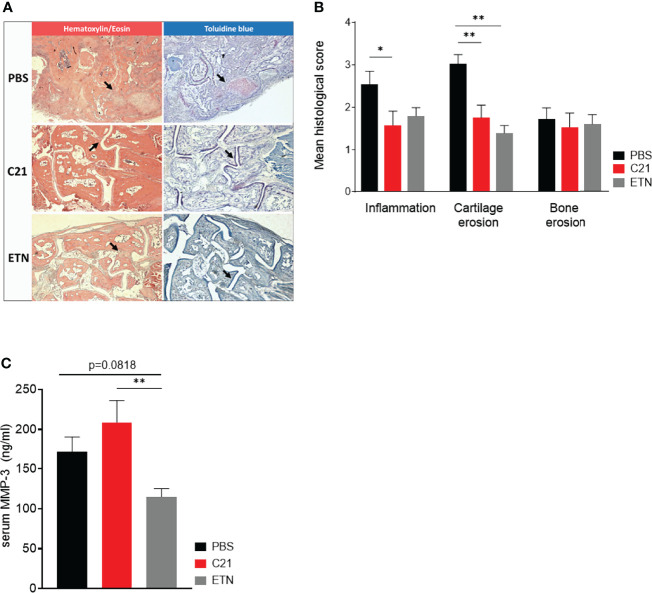
C21 treatment reduces inflammation and cartilage erosions, but did not alter serum MMP-3 concentrations. **(A)** Paraffin-embedded sections were stained with hematoxylin/eosin (HE), toluidine blue (TB) and tartrate-resistant acid phosphate (TRAP). Representative histopathological images of the early therapeutic C21 treatment are shown. HE (left column) and TB-stained (right column) hind paw sections of CII-immunised mice at day 47 upon PBS (upper row), C21 (middle row) and ETN treatment (lower row) are shown. Areas of inflammatory infiltrates **(Panel A,** left column**)** and cartilage damage **(Panel A,** right column**)** are indicated by arrows. Original magnification 5x. **(B)** The sections were evaluated for inflammation, cartilage erosions and bone erosions according to a graded scale from 0-3 and expressed as mean histological score. PBS (n=45), C21 (n=41), ETN (n=10). **(C)** Total MMP-3 concentrations were measured by ELISA in serum samples on day 47 of the early therapeutic approach. PBS (n=20), C21 (n=17), ETN (n=18). P*<0.05, P**<0.01. Values shown are the mean ± SEM. P-values were using the Kruskal-Wallis test followed by Dunn’s post hoc test for multiple comparisons.

To assess joint architecture, we histologically analyzed hind paws from the early therapeutic C21-intervention trial at day 47. The degree of inflammation, cartilage and bone damage was assessed in hematoxylin/eosin (HE) (inflammation), toluidine blue (TB) (cartilage) and tartrate-resistant acid phosphatase (TRAP) (bone) stained sections of each group (PBS, C21, ETN) by respective scores. Representative images of HE (left panel) and TB-stained (right panel) paraffin sections of hind paws are shown in **Figure 2A**. Histological assessment revealed reduced inflammation and cartilage breakdown upon C21 and ETN treatment compared to vehicle **(** **Figure 2B** **)**. The evaluation of TRAP-stained sections did not show differences in bone structure between the groups **(** **Figure 2B** **)**.

In RA and experimental arthritis, the family of MMPs plays not only an important role in the proteolytic cleavage of articular cartilage but also of the extra-cellular matrix (ECM) of the synovial joints (43). MMP-3 is known to degrade CII, the predominant form of collagen in articular cartilage (44), whereby MMP-3 concentrations are elevated in serum of humans and mice with active arthritis (45, 46). In our CIA experiments, there was a trend towards lower MMP-3 serum concentrations at day 47 in the etanercept group compared to the PBS group and the difference was statistically significant when comparing the etanercept group with the C21 group. Hence, daily injections of C21 in the early therapeutic treatment did not markedly alter MMP-3 concentrations, whereas etanercept slightly decreased MMP-3 concentrations **(** **Figure 2C** **).**

Taken together, C21-induced AT_2_R stimulation mediates anti-inflammatory actions resulting in reduced joint inflammation and the preservation of articular cartilage in CIA. A decrease in cartilage-degrading MMP-3 is most likely not a relevant mode of action in inhibition of inflammation and cartilage protection by C21.

### C21 Treatment Does Not Alter the Humoral Response Against Collagen Type II

**Figure 3:**
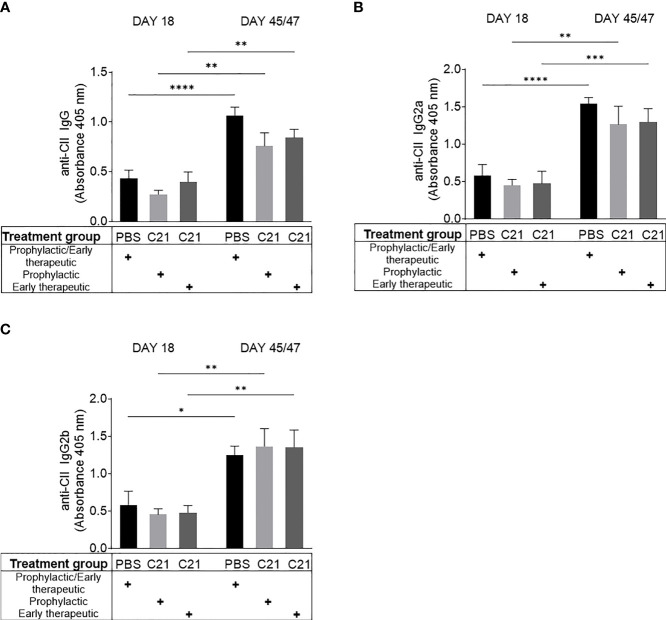
AT_2_R stimulation did not alter antibody production against collagen type II (CII). Sera were collected within the prophylactic and the early therapeutic intervention trial at two time points (day 18 and day45/47 after CII immunization). Circulating anti-CII-IgG **(A)**, anti-CII IgG2a **(B)** and anti-CII IgG2b **(C)** antibodies were determined by ELISA and absorbances at 405nm are shown. PBS (n=10) [black bars], prophylactic C21 treatment (n=9) [light grey bars], early therapeutic C21 intervention (n=10) [dark grey bars]. P*<0.05, P**<0.01, P***<0.001, P****<0.0001. Values shown are the mean ± SEM. P-values were calculated using two-way ANOVA with Bonferroni’s correction.

CII immunization evokes the production of anti-CII autoantibodies leading to immune complex-initiated joint damage (47, 48). We asked in this set of experiments whether AT_2_R stimulation by C21 modulates anti-CII antibody production in CII-immunized mice. Serum was collected at day 18 and day 45/47 post immunization and analyzed for total anti-CII IgG **(** **Figure 3A** **)**, anti-CII IgG2a **(** **Figure 3B** **)**, and anti-CII IgG2b **(** **Figure 3C** **)** by ELISA. During the course of the disease (day 18 *vs*. day 45/47), we observed a significant increase in anti-CII antibody levels in the PBS [black bars] as well as in the C21 groups [prophylactic (light grey bars) and early therapeutic (dark grey bars) treatment] **(** **Figures 3A–C** **)**. Moreover, at day 18 and day 47, anti-CII IgG, anti-CII IgG2a and anti-CII IgG2b antibody levels did not significantly differ between vehicle and C21-treated mice, neither to the prophylactic **(** **Figures 3A–C** **, C21)** nor to the early therapeutic approach **(** **Figures 3A–C** **, C21)**.

Altogether, C21 treatment did not alter the humoral immune response against CII. Hence, we conclude that anti-CII antibody production and consequently IC-formation, which eventually leads to the attraction of inflammatory immune cells into the joints, might not be markedly affected by C21.

### C21 Treatment of Activated Endothelial Cells Does Not Reduce Transendothelial Migration of Neutrophils *In Vitro*

**Figure 4:**
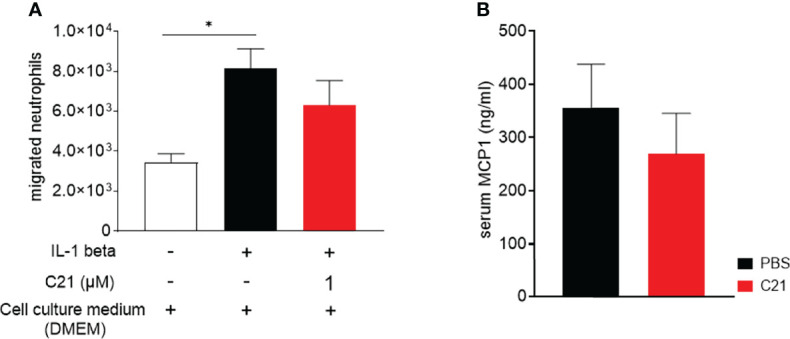
*In vitro* transmigration assay of human neutrophils through C21-treated and cytokine-activated endothelial cells and MCP-1 concentrations in sera of CIA mice. **(A)** Mesenteric lymph node endothelial cells (*mlENDs*) were treated with 1µM C21 or medium and stimulated with recombinant mouse IL-1 beta. The numbers of migrated neutrophils were counted. Experiments were performed in triplicates and performed three times each. P*<0.05. Values shown are the mean ± SEM. P-values were calculated by one-way ANOVA followed by Bonferronis’s correction for multiple comparisons. **(B)** MCP-1 concentrations were measured by ELISA in serum samples on day 47 of the early therapeutic approach. PBS (n=30), C21 (n=26). Values shown are the mean ± SEM. P-values were using the Kruskal-Wallis test followed by Dunn’s *post hoc* test for multiple comparisons.

Beside the role of pathogenic immune cells like macrophages/monocytes, neutrophils, T- and B cells in inflammatory arthritis (1, 5), endothelial cells are critically involved in the pathogenesis of arthritis by controlling the transmigration of leukocytes into the sites of inflammation (31, 49). An *in vitro* transwell migration assay was performed in order to investigate the impact of C21 on cytokine-activated endothelial cell function. IL-1 beta stimulated *mlEND* cells were incubated with C21 at a dose of 1µM or without the substance (medium control) and neutrophils migrated through the endothelium were counted. C21 did not significantly influence the number of migrated neutrophils compared to the medium control **(** **Figure 4A** **)**.

Furthermore, we investigated the concentrations of the MCP-1 [chemokine nomenclature: C–C motif chemokine ligand 2 (CCL2)] protein in the sera of CIA mice. MCP-1 orchestrates the migration and the infiltration of leukocytes into the sites of inflammation. In RA and experimental arthritis, increased levels of MCP-1 protein were found in the sera and synovial fluids (50). In the early therapeutic approach, we found similar concentrations of MCP-1 protein in the vehicle and C21-treated mice **(** **Figure 4B** **)** indicating the absence of direct effects of C21 on MCP-1 expression. To conclude, these data suggest that C21 does not induce anti-inflammatory properties by modulating endothelial cell function and the extravasation of leukocytes into the affected joints.

### Reduction of Immune Cells Expressing IL-1 Beta and IL-17A in Joints of C21-Treated CIA Mice

**Figure 5:**
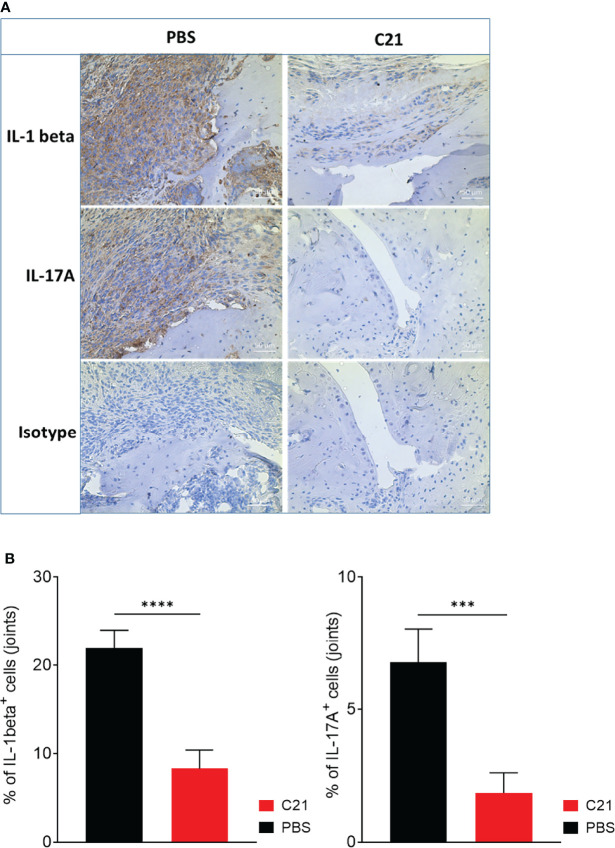
Reduced infiltrates of IL-beta and IL17A expressing cells in joints of CIA mice as a consequence of early therapeutic C21 treatment. **(A)** Paraffin-embedded sections of CIA hind paws were immunohistochemically stained for IL-1 beta, IL-17A and isotype control. Positive cells were stained in brown (3,3′-diaminobenzidine 4-HCl, DAB). Hematoxylin (blue) was used for counterstaining. Representative images are shown. Original magnification 20x. **(B)** Quantification of IL-1 beta and IL-17A stained joint sections. Sections of 4 randomly chosen mice of each group from one experiment were used and 3 fields per section were analyzed. ***= P < 0.001, P****<0.0001. Values shown are the mean ± SEM. P-values were calculated using the nonparametric Mann–Whitney U test.

The accumulation of immune cells in the joints promotes joint inflammation. Local IL-1 beta and IL-17A strongly contribute to progression of experimental and human arthritis (2, 8, 51, 52). To identify IL-1 beta and IL-17A expressing immune cells in joints of PBS and C21-treated CIA mice, immunohistochemical (IHC) stainings were performed using *3,3’-Diaminobenzidine* tetrahydrochloride (DAB) as substrate for the peroxidase labelled secondary antibodies. Representative images of IL-1 beta, IL-17A- and isotype control-stained paraffin-sections are shown in **Figure 5A**. Strong invasion of IL-1 beta **(** **Figure 5A**; left panel, upper row) and IL-17A **(** **Figure 5A**; left panel, middle row) expressing cells was found in the pannus-like tissue infiltrates of PBS treated CIA mice, whereas C21-treated mice exhibited only sparse areas of IL-1 beta^+^ immune cells **(** **Figure 5A**; right panel, upper row). IL-17A^+^ immune cells were virtually absent upon C21 treatment **(** **Figure 5A**, right panel, middle row). No DAB^+^ cells were detected in isotype control-stained paraffin-sections **(** **Figure 5A**, lower row). The percentages of IL-1 beta^+^ and IL-17A^+^ immune cells were assessed in the respective adjacent joint sections showing significantly lower percentages of IL-1 beta^+^ **(** **Figure 5B**, left panel) and IL-17A^+^ immune cells **(** **Figure 5B**, right panel) in C21-treated CIA mice compared to the control group.

In summary, the data suggest that the beneficial effect by C21 on arthritis might be a result of a modulated monocyte and T cell activation rather than a reduced immune cell migration.

### C21-Induced AT_2_R Stimulation Enhances the Numbers of Regulatory T Cells in CII-Immunized Mice

**Figure 6:**
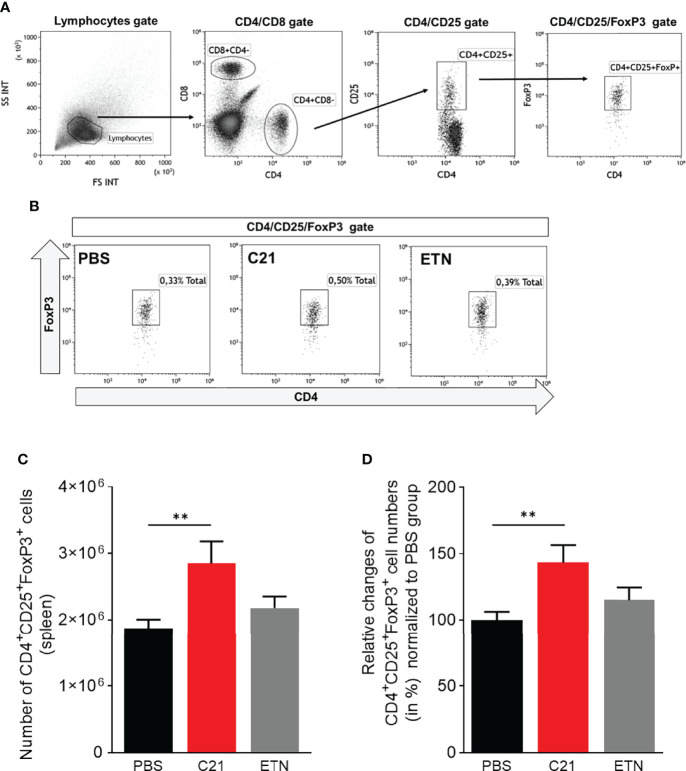
C21-induced AT_2_R stimulation raises the numbers of regulatory T cells in CIA in the early therapeutic intervention trial. Splenocytes from CIA mice were isolated at day 47 after CII-immunisation and stained with fluorochrome-conjugated antibodies against cell surface CD4, CD8, CD25 and intracellularFoxP3 and analyzed by flow cytometry. **(A)** Gating strategy to identify CD4^+^CD25^+^FoxP3^+^ Tregs. Lymphocytes were analyzed using forward scatter (FSC) and side scatter (SSC). Subgating of lymphocytes were performed resulting in CD8 versus CD4 dot plots followed by CD4 versus CD25 dot plots. Treg cells were enumerated by CD4^+^CD25^+^FoxP3^+^ Tregs. **(B)** Representative dot plots of each group (PBS, C21, ETN) are presented showing the frequencies (%) of CD4^+^CD25^+^FoxP3^+^ Tregs. **(C)** The absolute numbers of splenic CD4^+^CD25^+^FoxP3^+^ cells were calculated by using total spleen cell numbers. The results of one representative experiment are shown (PBS, n=10 mice; C21, n=8 mice; ETN, n=9 mice). P**<0.01. Values shown are the mean ± SEM. P-values were calculated using the Kruskal-Wallis test followed by Dunn’s *post hoc* test for multiple comparisons. **(D)** The summarized data of three independent CIA experiments were illustrated as relative changes (in %) normalized to the respective control group of each experiment (PBS = 100%) (PBS, n=32 mice; C21, n=26 mice; ETN, n=17 mice). P**<0.01. Values shown are the mean ± SEM. P-values were calculated by one-way ANOVA followed by Bonferroni’s multiple comparisons test.

An imbalance of Th subsets, in particular of the Th17/Treg cell ratio, is involved in the pathogenesis of arthritis (53, 54). Recently, a study showed the effect of C21 on the T cell response in experimental autoimmune encephalomyelitis (EAE), an animal model for multiple sclerosis (MS) (23). In order to investigate whether C21 has the capability to shift the Th17/Treg balance in CIA towards a more anti-inflammatory state, we isolated splenocytes at day 47 and stained for intracellular FoxP3 expression in CD4^+^CD25^+^ T cells, thereby identifying Tregs. A representative gating strategy for CD4^+^CD25^+^FoxP3^+^ Treg immunophenotyping by flow cytometry is presented in **Figure 6A**. Representative dot plots of CD4^+^CD25^+^FoxP3^+^ gates of each treatment group (PBS, C21, ETN) showing the frequencies (%) of CD4^+^CD25^+^FoxP3^+^ Tregs in respect to living splenocytes is presented in **Figure 6B**. The absolute numbers of CD4^+^CD25^+^FoxP3^+^ T cells per spleen were calculated as described and found increased upon C21 treatment. **Figure 6C** shows absolute numbers of Treg from one representative experiment. Due to the relative high variability of absolute cell numbers in between experiments, the summarized data of the three independent CIA experiments were expressed as relative changes (in %) compared to the respective PBS groups regarded as 100%. The relative percentages of CD4^+^CD25^+^FoxP3^+^ Tregs were significantly elevated in C21-treated mice in comparison to vehicle-treated animals **(** **Figure 6D** **)**. In ETN-treated CIA mice, the frequencies of CD4^+^CD25^+^FoxP3^+^ T cells were numerically slightly higher than in PBS-treated mice and lower than in C21-treated mice, however, both differences were not statistically significant.

These data suggest that C21 may induce an expansion of Treg cells, thereby controlling inflammation and improving CIA.

### *In Vitro*, C21 Promotes Treg Cell Differentiation and Inhibits Th17 and Th1 Cell Differentiation

**Figure 7:**
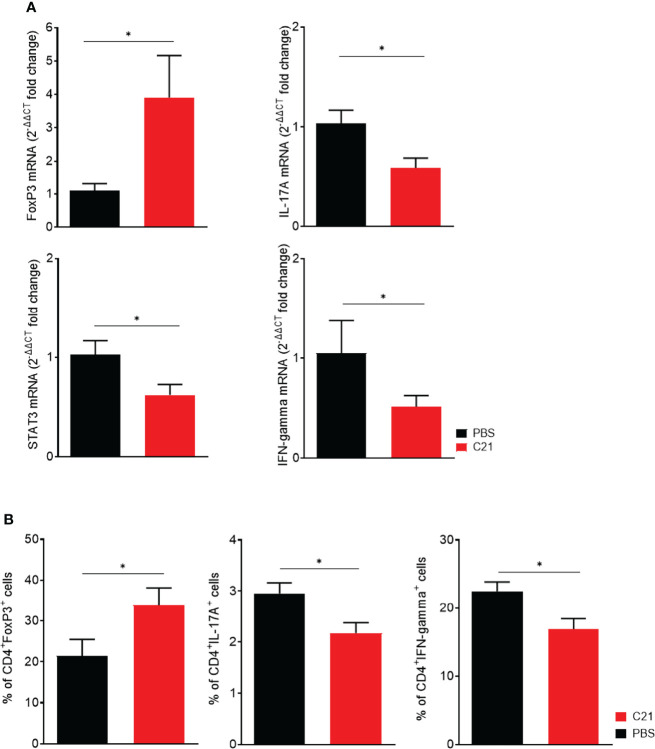
*In vitro*, the presence of C21 suppresses Th17 and Th1 cell differentiation and induces Treg cell differentiation. Naïve T cells (CD4^+^CD25^-^CD62L^+^) were isolated from C57BL6/J mice and polarized under Treg, Th17 and Th1 polarizing conditions for 6 days in the absence or presence of 1 µM C21. **(A)** Analysis of mRNA expression for FoxP3, IL-17A, STAT3 and IFN-gamma by real-time RT-PCR. Data were normalized for Hypoxanthin-Guanin-Phosphoribosyl-Transferase (HPRT) content. The PBS group was used as calibrator. Fold change expression was calculated by [2^(-ΔΔCT)]. Data are shown from three biological replicates. **(B)** Flow cytometric analysis of polarized T cells for intracellular FoxP3 (Treg), IL-17A (Th17) and IFN-gamma (Th1) in CD4^+^ T cells. Data from four biological replicates are shown. P* < 0.05. All data were expressed as mean values ± SEM. P values were calculated by the nonparametric Mann–Whitney U test.

Next, *in vitro* experiments were performed to corroborate the finding of increased Treg numbers in CIA upon C21 treatment. *In vitro*, naïve CD4^+^ T cells can be differentiated to Treg, Th17 and Th1 subsets by addition of specific polarizing cytokine cocktails (55). In the absence and the presence of C21 at a dose of 1µM, the mRNA expression levels of FoxP3, IL-17A, STAT3 and IFN-gamma were analyzed by real-time polymerase chain reaction (RT-PCR) in order to identify T cells with characteristics of Treg, Th17 or Th1 cells. In addition, the percentages of CD4^+^Foxp3^+^, CD4^+^IL-17A^+,^ and CD4^+^ IFN-gamma^+^ differentiated Th cell subsets were analyzed by flow cytometry. The presence of C21 during T cell differentiation significantly enhanced the expression of FoxP3 mRNA in Treg polarized cells compared to the medium control. The mRNA expression of IL-17A and STAT3 was significantly lower in Th17 differentiated cells upon C21 treatment. Moreover, IFN-gamma mRNA expression was significantly lower in C21-treated cells under polarizing Th1 conditions **(** **Figure 7A** **).** Consistent with the mRNA data, C21 upregulated the percentage of CD4^+^FoxP3^+^ cells in Treg polarizing cultures compared to controls **(** **Figure 7B** **)**. Furthermore, the percentage of CD4^+^IL-17A^+^ and CD4^+^IFN-gamma^+^ T cells was significantly suppressed upon C21 treatment **(** **Figure 7B** **)**.

Hence, C21-induced AT_2_R stimulation might alter the balance of Th subsets towards an anti-inflammatory milieu by expanding Tregs and suppressing Th1 and Th17 cells. These data suggest that C21 exerts its beneficial effects on CIA mainly by the induction of Treg and inhibition of Th1 and Th17 differentiation.

## Discussion

The main receptors for angiotensin, AT_1_R and AT_2_R, have opposing roles and biological actions, with the AT_2_R mediating a range of tissue protective effects (17, 24, 56–58). The synthesis of the non-peptide AT_2_R agonist C21 revolutionized AT_2_R research due to its very high affinity and selectivity for the AT_2_R and – in contrast to previously available peptide agonists - its metabolic stability and oral bioavailability (29, 57). A therapeutic effect of AT_2_R stimulation with C21 has been demonstrated in various preclinical disease models including experimental autoimmune encephalomyelitis (EAE), an animal model for multiple sclerosis (MS). In this model, AT_2_R stimulation protected the myelin sheaths in EAE by inhibiting microglia activation and T cell response (23).

The effects of C21 on experimental arthritis have not been investigated yet. In the current study, we provide evidence that C21 exerts beneficial effects on clinical manifestations and histopathology in CIA, a commonly used mouse model to study RA (39, 59).

For our study we used immunization of DBA/1J mice with heterologous collagen type II in complete Freund’s adjuvants, which induces autoantibodies to collagen type II, eventually leading to immune complex (IC) formation followed by complement activation, attracting neutrophils, monocytes/macrophages, T cells, B cells and mast cells into the joints (40, 60). Both, the prophylactic and the early therapeutic intervention (starting on day 20 after immunization) with C21 resulted in decreased arthritis severity and arthritis incidence compared to vehicle-treated mice. In the early therapeutic intervention trial, the therapeutic efficacy of C21 was comparable to the TNF-alpha antagonist etanercept, which is frequently used as potent biological agent in RA patients with insufficient response to conventional disease modifying drugs such as methotrexate (41). In RA, ETN is well tolerated and has a favorable safety profile. However, there is a risk of reactivation of latent tuberculosis as with other TNF antagonists (41). ETN acts as a decoy receptor and renders TNF biologically inactive. Thereby, ETN inhibits the multiple TNF-alpha-mediated pro-inflammatory effects such as the expression of adhesion molecules (E-selectin) and matrix-metalloproteines (MMP-3) (61). ETN was shown to ameliorate CIA by reducing the production of anti-CII IgG antibodies and has regulatory effects on the activation of B cells and the generation of memory B cells (42). In contrast to the findings of Wang et al. (42), C21 treatment did not lower the level of pathogenic anti-CII antibodies, neither in the prophylactic nor in the early therapeutic setting, indicating that C21 does not directly modulate B cell function. Moreover, therapeutic C21 administration resulted in a stronger inhibition of the early phase of arthritis than ETN, which might indicate a different mode of action in the early inflammatory phase of the disease. C21 is very well tolerated in animals, also we did not observe any overt adverse effects during our experiments. Currently, C21 is in early clinical development exhibiting a favorable safety profile.

In arthritis, synovial inflammation is eventually a result of transmigration of immune cells out of the blood vessels through the activated endothelium into the joint (49, 62, 63). Immunohistological examinations of the joints revealed significantly less IL-1 beta expressing cells and virtually absent IL-17A expressing cells in C21-treated CIA mice compared to controls, therefore indicating a strong anti-inflammatory and immune-modulatory effect of C21. IL-1 beta contributes to synovial inflammation and cartilage destruction, whereas IL-17A promotes inflammation by increasing the production of pro-inflammatory cytokines and the expression of receptor activator of nuclear factor-κB ligand (RANKL) (2, 64, 65). Moreover, a hallmark of synovial inflammation and cartilage turnover is the production of the active form of MMP-3 (66). MMPs such as MMP-1,-3, 9- and -13 play also an important role in the turnover of cartilage, synovial membrane, and connective tissue (43). We showed that the preservation of articular cartilage structure by C21 was independent of the increased MMP-3 expression suggesting a divergent mode of action compared to ETN. To get insights into the important step of leukocyte extravasation, we asked if C21 has the capability to directly modulate the function of endothelial cells, thereby possibly reducing the accumulation of inflammatory cells in the inflamed tissue. Sampson and colleagues reported evidence for protective effects of C21 in endothelial activation and adhesion of monocytes *in vitro* and under high-fat diet (HFD) in ApoE-/- mice, which was associated with an inhibition of ROS-induced NF-κB (nuclear factor kappa B) translocation (67). Furthermore, this group described that C21 did not modulate monocyte activation or macrophage polarization but did reduce the expression of TNFα and IL‐6 mRNA in M1 macrophages (67). With regard to the role of C21 in monocytes/macrophages, anti-inflammatory properties of C21 were also demonstrated in lipopolysaccharide (LPS)-activated THP-1 macrophages by increasing interleukin-10 production (35). In our experimental setting, we did not find decreased neutrophil transmigration through cytokine-activated endothelial cells in response to C21 treatment *in vitro*. Furthermore, serum MCP-1 concentrations were similar in PBS and C21-treated mice on day 47 after CII-immunization indicating a minor role of C21 on migration capacity or monocytes/macrophages functions. Together with our findings of an unaltered humoral immune response against CII in CIA, we assume that the C21-induced amelioration of CIA symptoms and tissue damage is rather to an alteration of the function of immune cells such as T cells, and not due to a reduction in transendothelial migration of leukocytes. This conclusion is supported by recent findings, which showed a C21-induced modulation of T cell function in EAE (23).

Autoimmune diseases such as RA and MS are characterized by an imbalance of pro-inflammatory Th17 cells and regulatory Treg cells (3, 4, 68). Targeting Th17 and Treg cells represents a potential therapeutic strategy in arthritis (69). In patients with autoimmune arthritis, Leipe et al. found elevated IL-17A levels in the peripheral circulation and synovial fluid that correlated with disease activity (70). Targeting the IL-17/IL-17R pathway represents a potential treatment target in RA and was already subject of clinical trials (71). A clinical phase II study testing the efficacy of the IL-17A neutralizing antibody secukinumab demonstrated that long-term treatment of RA patients, who were non-responders to DMARDs or biologics, resulted in a clinical improvement (12). However, a phase III study has questioned the benefit of secukinumab, because the primary endpoint [American College of Rheumatology response criteria 20 (ACR20) at week 24] was not met (13). Nonetheless, neutralization of IL-17A is an established treatment in patients with psoriatic arthritis and axial spondylarthritis. Frey and colleagues demonstrated a critical role of regulatory T cells in the model of antigen-induced arthritis (AIA). Treg depletion exacerbated AIA, whereas adoptive transfer of CD4^+^CD25^+^ Treg cells attenuated arthritis (72).

The discovery of agents that modulate the Th17/Treg differentiation axis towards a more anti-inflammatory state are of great therapeutic interest. Presently, monoclonal antibodies or small molecules that inhibit Th17 differentiation are under investigation for various autoimmune indications (73, 74). The induction of Treg cells might also represent a promising intervention in autoimmune diseases. The anti–TNF-alpha therapy with infliximab induces a boost of Treg cells in RA (75). In addition, the use of a low-dose IL-2 therapy is discussed controlling the inflammatory response *via* an induction of regulatory T cells (14, 76). Moreover, Helling et al. (77) developed the first humanized anti-CD4 mAb named tregaluzimab that selectively induces Treg activation. Although tregaluzimab was well tolerated by the patients in a clinical phase IIb study, treatment with this antibody did not show significant clinical efficacy in patients with active RA compared with placebo (78).

We found that C21 raises the numbers of CD4^+^CD25^+^FoxP3^+^ T cells in CIA, a fact that likely contributed to the improvement of arthritis. Recently, Hussain et al. showed protective effects of C21 on kidney ischemia by modulation of CD4 T cells towards the regulatory T cell phenotype (79).

Differentiation experiments of naïve Th cells into Th17, Th1 and Treg cells in the presence of C21 support the observed data in CIA. C21-induced AT_2_R stimulation promoted a shift towards an anti-inflammatory milieu by downregulating STAT3 and IL17A (Th17), as well as IFN-gamma (Th1) gene expression while upregulating FoxP3 in Treg polarizing conditions. STAT3 is critical in the regulation of joint inflammation by inducing Th17 differentiation (80, 81). Further, Liu and colleagues showed that STAT3 deletion in CD4^+^ T cells inhibits EAE development by blocking Th17 differentiation and promotes a shift towards anti-inflammatory T cell subsets (82). The exact molecular mechanism of C21-induced downregulation of STAT3 in T cells has not been clarified yet. The contribution of IFN-gamma in CIA pathology was described (83). However, this concept is controversially discussed since other researchers demonstrated that CIA and EAE are not attenuated in IFN-γ deficient mice or by blocking IFN-γ, indicating another T cell subset that is involved in the pathogenesis (84, 85). Finally, our data imply that the C21-induced clinical improvement in CIA might be predominantly regulated by the modulation of the Th17/Treg balance.

Our study has several limitations. First, we did not explore in detail the molecular and cellular mechanisms of action leading to Treg cell expansion upon C21 application. Second, we did not investigate the impact of C21 on Treg effector functions, which is beyond the scope of our study. Furthermore, it is worthwhile noting that we observed a trend towards reduced levels of circulating IL-1 but no changes in circulating IL-17A and IL-6 levels by C21 treatment. In addition, we detected numerically increased levels of circulating IL-10 at day 47 in C21-treated CIA mice compared to the PBS group (data not shown). However, these observations did not reach statistical significance which may be explained by the dynamic processes during the course of CIA in which cytokine secretion changes between early and late arthritis phases (86) and due to inter-individual differences in the cytokines release.

Of note, TGF-beta is known to promote the generation and function of Treg cells and can exert suppressive activities (87). Wan et al. reviewed in detail the negative (Yin) as well as the positive (Yang) roles for TGF-β and Treg cells in immune regulation (87). As pleiotropic cytokine, TGF-beta can also have immune-promoting functions such as proliferation, migration and others. Moreover, TGF-beta-1-signalling promotes tissue fibrosis (88). Several groups demonstrated the protective effect of C21 treatment on organ fibrosis in settings of cardiac and renal diseases. Inhibition of fibrosis was associated with an inhibition of inflammatory events (24). The relevance of C21 on fibrotic processes in arthritic joints remains to be elucidated. Hence, the influence of an altered turnover of the ECM and synovial connective tissue in CIA by C21 and its significance in relation to the observed beneficial effects in arthritis cannot be excluded. In our experiments concentrations of active TGF-beta 1 were below the detection limit in most serum samples at day 47 of CIA. Therefore, it remains unclear if C21 modulates the expression of TGF-beta in CIA.

As stated in the introduction, C21 is currently in Phase II/III clinical development idiopathic pulmonary fibrosis (www.vicorepharma.com). Therefore, it would be tempting to conduct a clinical trial to investigate the impact of C21 on arthritis severity and lung disease in RA patients with RA-associated interstitial lung disease (89, 90).

## Conclusions

Our study demonstrates that selective stimulation of the endogenous AT_2_R pathway by the non-peptidic Compound 21 has beneficial clinical and histological effects on CIA. Our data support the notion that AT_2_R stimulation acts immunomodulatory by increasing Treg cell numbers in the CD4 T-cell dependent model of CIA and regulates the Th17/Treg cell balance. Since preclinical experience and a recent clinical phase I and phase II studies with C21 point to very good tolerability of this first-in-class AT_2_R-agonist, C21 may represent a potential future drug to complement conventional RA medications.

## Data Availability Statement

The original contributions presented in the study are included in the article/supplementary material. Further inquiries can be directed to the corresponding authors.

## Ethics Statement

The animal study was reviewed and approved by Regierungspräsidium Freiburg Referat 35 Veterinärwesen, Lebensmittelüberwachung Bertoldstr. 43 79098 Freiburg, Germany.

## Author Contributions

Conceptualization: RV, BS, and US. Methodology: BS, GS, and US. Validation: RV, BS, and US. Investigations: BS and VV-E. Resources: GS and US. Data curation: BS, VV-E, and US. Writing—original draft preparation: BS. Writing and review and editing: BS, US, GS, TU, and RV. Visualization: BS and VV-E. Supervision: RV and US. Project administration: BS, US, and RV. Funding acquisition: GS, RV and US. All authors contributed to the article and approved the submitted version.

## Funding

This research was funded by the German Research Foundation (DFG) through the TRR 130 (project 12 to RV), the SFB 1160 (project 12 to RV and A. Triantafyllopoulou), the SFB 1181, project A01 to GS and Aline Bozec and the DFG Research group 2886 (PANDORA). In addition, the project was supported by the Erika Bürgy Fundation (Stiftung für die Region – Sparkasse Pforzheim Calw Treuhandstiftung Erika Bürgy Stiftung).

## Conflict of Interest

The authors declare that the research was conducted in the absence of any commercial or financial relationships that could be construed as a potential conflict of interest.

## Publisher’s Note

All claims expressed in this article are solely those of the authors and do not necessarily represent those of their affiliated organizations, or those of the publisher, the editors and the reviewers. Any product that may be evaluated in this article, or claim that may be made by its manufacturer, is not guaranteed or endorsed by the publisher.
